# Adverse Effects of the Apolipoprotein E ε4 Allele on Episodic Memory, Task Switching and Gray Matter Volume in Healthy Young Adults

**DOI:** 10.3389/fnhum.2017.00346

**Published:** 2017-06-29

**Authors:** Jianfei Nao, Hongzan Sun, Qiushi Wang, Shuang Ma, Shuo Zhang, Xiaoyu Dong, Ying Ma, Xiaoming Wang, Dongming Zheng

**Affiliations:** ^1^Department of Neurology, Shengjing Hospital of China Medical UniversityShenyang, China; ^2^Department of Radiology, Shengjing Hospital of China Medical UniversityShenyang, China

**Keywords:** executive function, memory, magnetic resonance imaging, apolipoprotein E, polymorphism

## Abstract

Many studies have shown that healthy elderly subjects and patients with Alzheimer’s disease (AD) who carry the *apolipoprotein E* (*ApoE*) ε4 allele have worse cognitive function and more severe brain atrophy than non-carriers. However, it remains unclear whether this *ApoE* polymorphism leads to changes of cognition and brain morphology in healthy young adults. In this study, we used an established model to measure verbal episodic memory and core executive function (EF) components (response inhibition, working memory and task switching) in 32 *ApoE* ε4 carriers and 40 non-carriers between 20 years and 40 years of age. To do this, we carried out an adapted auditory verbal learning test and three computerized EF tasks. High-resolution head magnetic resonance scans were performed in all participants and voxel-based morphometry (VBM) was used for image processing and analysis. Multivariate analysis of variance (ANOVA) performed on memory measures showed that the overall verbal episodic memory of *ApoE* ε4 carriers was significantly worse than non-carriers (Wilk’s *λ* = 4.884, *P* = 0.004). No significant differences were detected in overall EF between the two groups. *Post hoc* analyses revealed group differences in terms of immediate recall, recognition and task switching, which favored non-carriers. VBM analysis showed gray matter (GM) bilateral reductions in the medial and dorsolateral frontal, parietal and left temporal cortices in the carrier group relative to the non-carrier group, which were most significant in the bilateral anterior and middle cingulate gyri. However, these changes in GM volume were not directly associated with changes in cognitive function. Our data show that the *ApoE* ε4 allele is associated with poorer performance in verbal episodic memory and task switching, and a reduction in GM volume in healthy young adults, suggesting that the effects of *ApoE* ε4 upon cognition and brain morphology exist long before the possible occurrence of AD.

## Introduction

The ε4 allele of *apolipoprotein E (ApoE)* is the most clearly defined genetic risk factor for sporadic Alzheimer’s disease (AD). Carriers of the ε4 allele are not only at an increased risk of AD but also have a significantly earlier onset age of AD (Shaw et al., 2007; Chang et al., 2016). However, it is still not clear how *ApoE* gene polymorphism is involved in the pathogenesis of AD. In recent years, many studies have found that brain atrophy in AD and mild cognitive impairment (MCI) patients carrying *ApoE* ε4 is more prevalent than in non-carriers (Basso et al., 2006; Filippini et al., 2009b; Schuff et al., 2009; Spampinato et al., 2011), and their cognitive decline is also more significant (Okonkwo et al., 2010; Whitehair et al., 2010; Wattmo et al., 2011; Vos et al., 2013; De Beaumont et al., 2016). However, the effects of* ApoE* gene polymorphisms upon brain morphology and cognitive function are not limited to AD patients; brain atrophy and cognition impairment associated with *ApoE* ε4 carriers were also found in studies of healthy elderly subjects (Honea et al., 2009; Crivello et al., 2010; Lu et al., 2011) and in non-AD patients with neurological disorders, such as frontotemporal dementia (Boccardi et al., 2004; Agosta et al., 2009), Parkinson’s disease (Mata et al., 2014) and HIV infection (Wendelken et al., 2016). Therefore, we hypothesized that the effects of *ApoE* polymorphism on brain morphology and function may have no specific relationship with the pathophysiological process of AD, but is more likely to affect brain growth and development, aging and pathological repair through specific mechanisms, which increases the risk of neurological diseases in *ApoE* ε4 carriers.

In this study, we therefore recruited healthy young adults as the study population to investigate whether the effects of the *ApoE* ε4 allele upon brain morphology and brain function are apparent at an age when brain development has matured. In this regard, there have been a few studies performed in young populations. However, unlike the studies in healthy elderly people, which have consistently shown that *ApoE* ε4 carriers performed significantly worse in a variety of cognitive tasks (Wisdom et al., 2011) and had reduced gray matter (GM) volume in the hippocampus and other related brain regions compared to non-carriers (Honea et al., 2009; Crivello et al., 2010; Lu et al., 2011), the studies performed in young populations arrived at various conclusions. For example, the cognition of young *ApoE* ε4 carriers was reported to be either inferior (Acevedo et al., 2010; Chang et al., 2016), superior (Mondadori et al., 2007; Jochemsen et al., 2012; Rusted et al., 2013) or equivalent (Reiman et al., 2004; Taylor et al., 2011; Bunce et al., 2014; Jack et al., 2015; Matura et al., 2016) to non-carriers. The differences in these conclusions suggest that this issue is far from being solved.

Episodic memory impairment is the earliest and most significant brain function involved in AD. The impairment in executive function (EF) also occurs early in AD (Zheng et al., 2012) and is one of the main reasons underlying the inability of AD patients to work and live unassisted. Therefore, this study chose to specifically assess episodic memory and EF. As EF is not a single component brain function, we use computerized EF tasks to assess the key components of EF based on Miyake’s theoretical model of EF, which includes response inhibition, working memory and task switching (Miyake et al., 2000). Using latent-variable analysis,Miyake et al. (2000) verified that although the three key EFs were moderately correlated with one another, they were still distinct. In our previous study, we demonstrated that this type of evaluation is more likely to detect slight changes in EF than the pen-and-paper version (Zheng et al., 2012). High-resolution head magnetic resonance scans and voxel-based morphometry (VBM) analysis was used to explore changes of brain morphometry related to *ApoE* ε4. We hypothesized that there would be mild but significant differences in these cognitive functions and brain morphology when compared between *ApoE* ε4 carriers and non-carriers which would favor the latter.

## Materials and Methods

### Participants

A total of 72 young, healthy volunteers were enrolled in this study, all of which came from our previous investigative study of a young population (*n* = 246) in which *ApoE* genotyping had already been performed. The inclusion criteria for the participants in the previous study included the following: age between 20 years and 40 years old; no history of neurological disorders, mental illness or brain trauma; no daily alcohol consumption or smoking habits; no history of hypertension or diabetes; and a Montreal Cognitive Assessment (MoCA) score within the normal range. The method for *ApoE* genotyping is described below. All 32 ε4 carriers (*ApoE* ε3/ε4 or ε4/ε4 genotypes) in the previous study were recruited into this present study and constituted the ε4+ group. Forty *ApoE* ε3/ε3 participants with matching age, gender and education level were included in the ε4− group. *ApoE* ε2 carriers were not included in either group because the function of ε2, and the interactions between ε2 and other alleles, are less understood and because the frequency of the ε2 allele was very low in this population. Participants were required to take verbal episodic memory and EF tests and undergo a head magnetic resonance imaging scan. The study was approved by the Ethics Committee of Shengjing Hospital, China Medical University. Written informed consent in accordance with the Declaration of Helsinki was obtained from all participants. The clinical features of the two groups, including age, gender, education level, handedness (determined by the Chinese version of the Edinburgh Hand Questionnaire) and MoCA score, are listed in Table 1.

**Table 1:** Demographic characteristics and MoCA scores of ε4+ and ε4− groups.

Group	ε4+ (*n* = 32)	ε4− (*n* = 40)	*t* or *χ*^2^	*P*
Sex (male/female)	12/20	16/24	0.047	0.829
Age, y	27.5 (5.0)	28.1 (5.3)	−0.487	0.628
Education, y	16.6 (2.7)	16.9 (2.8)	−0.429	0.669
Right-handedness	30	38	0.053	0.818
MoCA score	29.5 (0.7)	29.7 (0.5)	−1.432	0.158

*Data represent the mean (standard deviation). MoCA, Montreal Cognitive Assessment*.

### *ApoE* Genotyping

For genotyping, 2 ml of elbow vein blood was collected and placed in a blood collection tube containing ethylene diamine tetraacetic acid (EDTA). Leukocytes were then isolated for *ApoE* genotyping. The genomic DNA of white blood cells, which was used as a polymerase chain reaction (PCR) template after dissolving in distilled water, was extracted using the phenol/chloroform method.

Primer Premier 5.0 software (Premier Biosoft, Palo Alto, CA, USA) was used to design the specific amplification primers for *ApoE*. The upstream primer (*ApoE*-F) was 5′-GCCCCGTTCCTTCTCTCCCTCTT-3′, and the downstream primer (*ApoE*-R) was 5′-CCGGCTGCCCATCTCCTCCATC-3′. The size of the PCR product was 647 bp. The reaction consisted of 12.5 μl of 2× PCR Mix (Promega, Madison, WI, USA), 1 μl of each primer (10 μM), and 1 μl of template DNA (30–60 ng/μl), with double distilled water added to a total volume of 25 μl. The PCR conditions were 95°C for 2 min, then 30 cycles of 95°C for 15 s, 58°C for 30 s and 72°C for 40 s, followed by a 5-min extension at 72°C. Specific amplification products were resolved by 1.5% agarose gel electrophoresis. Sequencing analysis was carried out using an ABI 3730XL sequencer (ABI, Waltham, MA, USA) with *ApoE*-F as sequencing primers; Chromas software (Technelysium, South Brisbane, Australia) was used to analyze the results. The corresponding alleles result in two different amino acid combinations at positions 112 and 158: ε2 (112Cys/158Cys), ε3 (112Cys/158Arg) and ε4 (112Arg/158Arg). The SNP codes of these two loci are rs429358 (amino acid 112, C/T) and rs7412 (amino acid 158, C/T).

### Verbal Episodic Memory

We used an adapted Chinese version of the World Health Organization University of California-Los Angeles Auditory Verbal Learning Test (WHO-UCLA AVLT; Maj et al., 1994) to examine verbal episodic memory. We extended the 15 words conventional AVLT to 20 words to increase difficulty. The selected words were read aloud three times, and each time, the participants were asked to memorize as many of them as possible; the total number of words correctly recalled within three times was calculated as the immediate recall score. After a 30-min delay, a recall trial and recognition test was conducted with the number of correctly recalled words as a measure of long delayed recall. For the recognition test, subjects were presented with a list of the 20 studied words and 20 non-studied foils and were asked to circle all words previously given. The scores were calculated as the number of correctly recalled learned words minus the number of marked non-studied foils.

### Core EF Components

The core EF components, which are response inhibition, working memory and task switching based in the EF model developed by Miyake et al. (2000), were measured with computerized tasks. EF tasks were intentionally selected to be sensitive and specific for the evaluation of a single core EF component: working memory was assessed with a “keep-track” task, response inhibition was assessed with a “stop-signal” task, and task switching was assessed with a “more-odd shifting” task. These tasks were programmed with E-prime 2.0 (Psychology Software Tools, Pittsburg, PA, USA). The responses were logged using buttons or vocal keys of the E-prime serial response box. As the same tasks were used, and described, in our previous studies (Zheng et al., 2012, 2014), only the basic designs of these tasks are introduced below. All participants were individually tested. The order of task administration was fixed for all participants (i.e., the more-odd shifting task, then the stop-signal task, and finally the keep track task). All participants received one 5-min practice session for each task prior to the formal test. There was a 3-min rest period between tasks. The entire testing period lasted approximately 1 h.

#### Keep Track Task

During each trial, the participants were first shown three target categories at the bottom of a computer screen. Then, a list of two-character Chinese words from four possible categories (i.e., animals, countries, plants and relatives) was serially presented in a random order for 1500 ms each, while the target categories remained at the bottom of the screen. The task was to remember the last word presented in each of the target categories and then to write down these words at the end of the trial. Three trials, consisting of 12, 16 or 20 words, were presented twice in a random order. One point was awarded for each correctly recalled word, with a total possible score of 18. The total score was the dependent measure.

#### More-Odd Shifting Task

A series of numbers (1–4 or 6–9) was displayed at the center of the screen. Each number appeared for 1000 ms. There were two conditions in the task: (1) when the number was red, the participants were required to say “big” as quickly as possible if the number appearing on the screen was greater than five and “small” if the number was less than five; and (2) when the number was green, the participants were required to say “odd” or “even” depending on the parity of the number. In the shifting block (S), which consisted of 48 trials, the participants regularly alternated between the two conditions, switching from one to the other every two trial intervals. Thus, the shifting block consisted of 23 switch trials and 25 non-switch trials. The control block (C) consisted of 24 trials of 1 condition and did not require a switch. The reaction times (RTs) were measured using a vocal key, and a tape recorder was used to record the answers. The participants were required to finish two shifting blocks and four control blocks (two blocks of each condition) in the order C-C-S-S-C-C. The switch cost was the difference between the average RTs of the switch trials in the shifting blocks and the average RTs of the non-switch trials in the control blocks.

#### Stop-Signal Task

In the Go trial, the participants were instructed to press a button as soon as possible when they saw the “go” signal (a circle). The circle disappeared when the button was pressed, or after 1000 ms had passed without a response, whichever came first. On the Stop trial, a “stop” signal (a cross) appeared shortly after the “Go” signal. The participants were instructed not to press the button in trials with a “stop” signal. In every four trials, one Stop trial and three Go trials were presented in a random order. A staircase-tracking algorithm was used to modify the time interval between the “stop” and “go” signals according to the responses of the participants. Using this algorithm, approximately 50% of all stop trials could be inhibited by participants, which yielded accurate estimates of stop signal RT. The formal test consisted of two blocks of 100 trials each.

### Magnetic Resonance Imaging

Magnetic resonance imaging scans were obtained using a Philips Intera Achieva 3.0 Tesla scanner with an eight-channel brain phased array coil. The high resolution T1-weighted images were acquired using a 3D Turbo Field Echo sequence with the following parameters: TR/TE/flip = 9.5 ms/4.6 ms/20°; acquisition matrix = 256 × 256 mm^2^; field of view = 220 × 220 mm^2^; slice thickness = 1.2 mm (182 horizontal slices).

### Voxel-Based Morphometry

SPM8 (Wellcome Trust Centre for Neuroimaging, London, UK^1^) running on Matlab 7.5.0 (Mathworks, Natick, MA, USA) was used for image processing. Each individual’s structural image was first co-registered to an ICBM152-space (i.e., Montreal Neurological Institute [MNI] space), and an average template was distributed with SPM8 using normalized mutual information. Structural images were then segmented into tissue classes using the “new segment” option of SPM8. By increasing the number of tissue classes and using less age-biased templates, the “new segment” provided a more accurate segmentation of brain tissue compared with the previous unified segmentation method of SPM5. Next, the DARTEL toolbox was used to derive a set of group-specific templates following a method described in the SPM8 manual in its standard version. The flow fields described the transformation from each native GM image to the template, which were then applied to each participant’s GM image. The DARTEL template was registered to the tissue probability maps using an affine transformation to transform the template-space images into the MNI space, and this transformation was then incorporated into the warping process. After warping, the segmented images were modulated using the Jacobian determinants derived from spatial normalization. These normalized and modulated images were then smoothed with an 8-mm full-width at half-maximum smoothing kernel for final statistical analyses.

### Statistical Analysis

SPSS for Windows version 11 (SPSS Inc., Chicago, IL, USA) was used for statistical analysis. *P*-values less than 0.05 were considered statistically significant. *T*-tests and χ^2^ tests were used to compare demographic features. Two multivariate analysis of variance (MANOVA) were performed respectively on the memory and EF measures to compare the overall verbal episodic memory and EF between the two groups. *Post hoc* analyses using analysis of variance (ANOVA) were conducted to examine the between-group differences on individual measures. The critical α level was adjusted to 0.017 (0.05/3) with Bonferroni correction in the *post hoc* comparisons. Due to the small sample size, Cohen’s *d* was calculated to determine the effect sizes of the comparisons.

In VBM, regional GM differences between the *ApoE* ε4+ group and ε4− groups were assessed using a two-sample *t*-test implemented in the general linear model approach of SPM8. Statistical threshold for this analysis was set at *P* < 0.05, family-wise error (FWE) corrected for multiple comparisons and a spatial extension of 10 voxels. A less stringent threshold of *P* < 0.001 (uncorrected) was used if the previous analysis did not reveal many significant brain regions. Age, educational level and total intracranial volume (TIV) were included as covariates of no interest to avoid false positive findings caused by potential confounding factors that are known to affect brain morphology. TIV was calculated using SPM8 from the unsmoothed, modulated GM, white matter and cerebrospinal fluid images obtained from each patient. Data were displayed using the xjView tool box^2^.

If significant differences were found in both brain function indicators and brain morphology between the ε4+ and ε4− groups, a multiple regression model in VBM was utilized for correlation analysis between differences in brain function and brain morphology. All multiple regression analyses were limited to the areas of significant GM volume reduction.

## Results

### Characteristics of the Participants

The demographic characteristics of the *ApoE* ε4+ and the *ApoE* ε4− groups are presented in Table 1. There were no significant differences in age, sex, educational level or handedness between the two groups. Furthermore, the MoCA scores did not differ between the two groups (*t* = −1.432, *P* = 0.158).

### Performance on AVLT and EF Tasks

The AVLT and EF task results of the two groups are given in Table 2. MANOVA, carried out on three memory measures, showed that the overall verbal episodic memory of *ApoE* ε4 carriers was significantly worse than non-carriers (Wilk’s *λ* = 4.884, *P* = 0.004), while MANOVA carried out on EF task measures was not significant (Wilk’s *λ* = 2.438, *P* = 0.072). ANOVA revealed significant group differences in the scores of total immediate recall (*F* = 6.434, *P* = 0.013, Cohen’s *d* = 0.586), recognition (*F* = 11.471, *P* = 0.001, Cohen’s *d* = 0.774) and task-switching ability (*F* = 6.254, *P* = 0.015, Cohen’s *d* = 0.599), which favored the ε4− group. Although other parameters in the ε4+ group were also worse than the ε4− group, these were not statistically significant.

**Table 2:** Mean (and standard deviation) scores and group differences in memory and EF measures between the two groups.

Task	Measure	*ApoE*ε4+ Group	*ApoE*ε4− Group	*F*	*P*	Cohen’s *d*
Keep track task	Total score	7.2 (1.8)	7.4 (1.6)	−0.397	0.693	0.117
Stop-signal task	Stop signal reaction time (ms)	255.0 (55.5)	239.5 (45.3)	1.303	0.197	0.306
More-odd shifting task	Switch cost (ms)	230.9 (85.9)	174.2 (102.8)	2.501	0.015	0.599
AVLT	Total immediate recall	35.5 (8.3)	39.8 (6.2)	−2.454	0.017	0.586
	Long delayed recall	15.0 (4.1)	16.1 (2.7)	−1.366	0.178	0.316
	Recognition	16.3 (2.6)	18.0 (1.7)	−1.718	0.002	0.774

*Key: EF, executive function; AVLT, Auditory Verbal Learning Test*.

### GM Differences between the Two Groups

**Figure 1:**
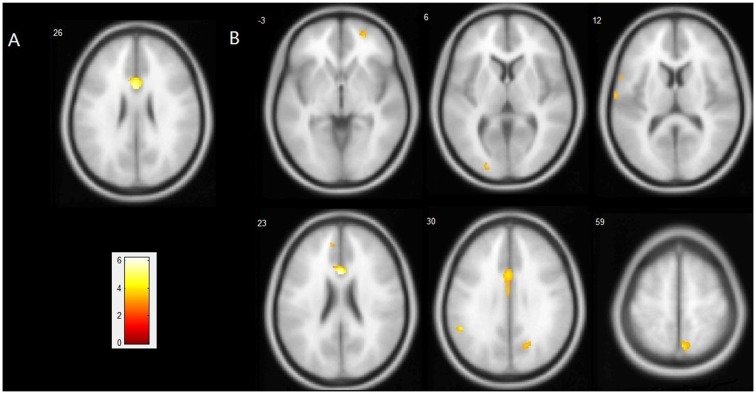
Regions showing a significant reduction of gray matter (GM) in the *ApoE*ε4^+^ group compared with the *ApoE*ε4^−^ group. **(A)** Statistical map threshold at *p* < 0.05 family-wise error (FWE) corrected for multiple comparisons and more than 10 voxels. **(B)** Statistical map threshold at *p* < 0.001 (uncorrected) and more than 10 voxels. The left side of the image represents the left hemisphere of the brain. Slice positions are indicated in Montreal Neurological Institute (MNI) coordinate *z* in the upper left corner of each slice. The MNI coordinates and anatomical descriptions are presented in detail in Table 3.

Compared with the ε4− group, the ε4+ group showed a significant reduction in GM volume in the bilateral anterior and middle cingulate gyri (MNI coordinates: *x* = −2, *y* = 19, *z* = 26, volume: 2086 mm3, BA24/23) with *p* < 0.05 FWE corrected for multiple comparisons and a spatial extension of 10 voxels. With a less stringent threshold of *p* < 0.001 (uncorrected), more brain regions distributed bilaterally in the frontal, temporal and parietal lobes were evident by VBM analysis, in which the ε4+ group had a relatively smaller GM volume than the ε4− group (Figure 1, Table 3). Within these brain regions, we performed three VBM multiple regression analyses in the ε4+ group using the scores of total immediate recall, recognition and task-switching individually. However, no brain regions were found to be responsible for the changes of these cognitive functions in the ε4+ group identified by multiple regression analyses. Compared with the ε4+ group, no brain regions in the ε4− group were found with a smaller GM volume.

**Table 3:** Atrophic brain regions in the *ApoE*ε4+ group vs. the *ApoE*ε4− group.

Anatomical structure	MNI coordinates			Volume (mm^3^)	*t*
	*X*	*Y*	*Z*
*p* < 0.05 FWE corrected for multiple comparisons:
L/R anterior/middle cingulate gyrus (BA 24/23)	−2	19	26	2086	6.25
*p* < 0.001 uncorrected:
R Precuneus (BA 7/31)	15	−71	35	820	4.70
R Precuneus (BA 7)	5	−66	59	581	4.08
L Supramarginal Gyrus (BA 40)	−53	−47	30	196	4.12
R Superior Frontal Gyrus (BA 10)	26	53	−3	250	3.79
L Medial Frontal Gyrus (BA 9)	−7	40	23	51	3.60
L Middle Occipital Gyrus (BA 18)	−21	−93	6	114	3.61
L Transverse Temporal Gyrus (BA 42)	−65	−12	12	122	3.59
L Inferior Frontal Gyrus (BA44)	−57	8	12	81	3.28

*Statistical map threshold at different *p* level and more than 10 voxels; 1 voxel = 1.5 mm × 1.5 mm × 1.5 mm. Key: BA, Brodmann area; L, left; MNI, Montreal Neurological Institute; R, right*.

## Discussion

Although many studies have consistently shown that, in both AD and MCI patients, the cognitive function of *ApoE* ε4 carriers is worse than non-carriers and that carriers suffered faster recession of cognition (Okonkwo et al., 2010; Whitehair et al., 2010; Wattmo et al., 2011; Risacher et al., 2013; Vos et al., 2013; De Beaumont et al., 2016), there have been no definitive conclusions regarding the effects of *ApoE* polymorphisms upon cognitive functions in the normal population, perhaps due to the confounding effect of age. Most studies in healthy elderly people have shown that *ApoE* ε4 carriers performed significantly worse on measures testing a range of neurocognitive functions. The meta-analysis by Wisdom et al. (2011) which included 77 studies with a total of 40,942 subjects, consisting of mainly elderly people with normal cognitive function, showed that *ApoE* ε4 exerted adverse effects on a range of neurocognitive functions and also that the difference in cognitive functions between *ApoE* ε4 carriers and non-carriers increased with age. However, in regard to studies in children, young and middle-aged adults, significant controversy remains. Some researchers argue that the relationship between the *ApoE* ε4 allele and cognition across the life span is an example of antagonistic pleiotropy, meaning that the *ApoE* ε4 allele may be beneficial at an earlier age and may become a risk factor for cognitive decline in later life (Tuminello and Han, 2011). This opinion arises from several studies which showed that healthy young adults and children carrying the ε4 allele exhibit better cognitive performance relative to non-ε4 carriers (Mondadori et al., 2007; Jochemsen et al., 2012; Rusted et al., 2013). However, the antagonistic pleiotropy hypothesis of *ApoE* ε4 upon cognition across the life span has been frequently doubted as there are a number of studies in which the cognition of young *ApoE* ε4 carriers were reported to be either inferior (Acevedo et al., 2010; Chang et al., 2016) or equivalent (Reiman et al., 2004; Taylor et al., 2011; Bunce et al., 2014; Jack et al., 2015; Matura et al., 2016) to non-carriers.

Although our current study cannot put an end to the above controversy, our data do provide new evidence to support the adverse effects of *ApoE* ε4 upon cognition in young populations. Our data showed that the verbal episodic memory of young *ApoE* ε4 carriers was inferior to non-carriers. Although the overall EF was not significantly affected by *ApoE* ε4, one of the key components of EF, task switching, was adversely affected by this allele. These findings indicated that the effects of *ApoE* gene polymorphism on some main cognitive domains had already emerged in young individuals. A recently published study by Chang et al. (2016) further showed that EF, working memory and attention in children with ε4/ε4 and ε4/ε2 genotypes were worse than children with other genotypes, which is similar to the findings of this present study in young adults. Collectively, these studies suggest that *ApoE* polymorphisms may affect brain maturity and development through certain mechanisms which can result in cognitive differences in early life. These mechanisms therefore warrant further study.

It should be noted that although the effect of *ApoE* gene polymorphism upon cognitive function exists in the young population, the extent of this effect is relatively small. In this study, we used an adapted AVLT with a higher degree of difficulty and three computerized EF tasks which can specifically detect each core component of EF, which can, to some extent, reduce a possible ceiling effect caused by using clinical assessments to evaluate cognition in well-educated young adults and the mutual compensation between different cognitive functions. With particular regard to the evaluation of EF, the three core components of EF were evaluated separately and quantitatively according to Miyake’s EF model. Despite this enhanced sensitivity, the two groups differed only in part of the memory, and EF measures, and none of these effects were extensive, indicating that both the cognitive domains affected by *ApoE* ε4, and the effect of *ApoE* ε4 upon these cognitive domains, were limited in young adults. The reason why only task switching was affected among the three key EF components may be related to the increased number of cognitive processes (inhibition of task-sets and top-down control of the task-set) and brain areas involved in this process (Aron et al., 2004) compared with other EF components such as response inhibition, which increases the chances of task switching recruiting an area affected by pathological changes. The limited effect size and the use of usual clinical cognitive assessment tools that are not sensitive enough may at least partially explain why some previous studies have failed to show any cognitive differences between young *ApoE* ε4 carriers and non-carriers. This small effect will likely increase with age and lead to marked differences in cognition between carriers and non-carriers in later life (Wisdom et al., 2011). Furthermore, the small effect of *ApoE* ε4 on cognition and brain structure at this young age may partially account for the negative VBM correlation analysis in this study.

Some neuroradiological studies have shown structural and functional differences between young *ApoE* ε4 carriers and non-carriers and favor the latter, which could be the basis of the lower cognitive function of young *ApoE* ε4 carriers. Similar to studies in AD patients, a PET study performed in young adults showed that the glucose metabolic rate of the cingulate gyrus, parietal, temporal and frontal cortex of young adults carrying *ApoE* ε4 was significantly reduced compared with non-carriers. This suggests that *ApoE* ε4 carriers have functional brain abnormalities in young adulthood, several decades before the possible onset of dementia (Reiman et al., 2004). Many functional magnetic resonance studies have also shown that young *ApoE* ε4 carriers need to mobilize a wider brain area than non-carriers when performing cognitive tasks (Filippini et al., 2009a; Dennis et al., 2010; Chen et al., 2013). The wider activation of brain areas in *ApoE* ε4 carriers could be evidence of less efficient brain networks which need to involve more brain areas to accomplish the same task. The over-activity of brain function in young ε4 carriers is disproportionately reduced with age (Filippini et al., 2011), and healthy middle-aged and elderly ε4 carriers begin to show reduced functional brain activity and in regions pertinent to AD (Lind et al., 2006; Brown et al., 2011). The change in activation patterns across life span suggests a possible subclinical impairment of cognition and the occurrence of a cognitive compensation mechanism in young *ApoE* ε4 carriers, which is weakened by aging. The current VBM study showed that young healthy *ApoE* ε4 carriers have a lower GM volume in multiple brain areas compared with non-carriers. There are only a limited number of similar studies conducted in younger subjects, and the conclusions of these studies were inconsistent. Using various radiological indexes and analytic methods, some studies showed *ApoE* ε4-related GM and white matter volume reductions (Shaw et al., 2007; Alexopoulos et al., 2011; Alexander et al., 2012; Dean et al., 2014; Chang et al., 2016), while others did not (Mondadori et al., 2007; Khan et al., 2014; Matura et al., 2014; Dell’Acqua et al., 2015; Gonneaud et al., 2016). Although a greater number of studies, with more participants, using consistent methods and inclusion criteria, are still needed to further clarify the conflicting results evident in previous studies, our present study of young adults, along with studies in children and adolescents (Shaw et al., 2007; Chang et al., 2016), and even infants (Dean et al., 2014), strongly indicates the continuous negative influence of the *ApoE* ε4 allele on brain growth and development. The large cohort study of Khan et al. (2014) showed that brain structures predominantly affected by AD, such as the hippocampus, were not affected by the ApoE polymorphism in young adults. Moreover, the study performed in AD and frontotemporal dementia patients showed ε4 carrier status was associated with more severe brain atrophy in disease-specific regions compared with noncarriers: medial temporal atrophy was greater in the AD carrying the ε4 allele, frontal lobe atrophy in the FTD carrying the allele (Agosta et al., 2009). Altogether, these structural and functional imaging studies suggest that the effects of *ApoE* polymorphism upon the central nervous system may not be limited to AD-related pathological processes, but may also affect the growth, development, aging and pathological repair capacity of the nervous system, which can lead to susceptibility to certain central nervous system diseases, such as AD.

Another strong line of evidence supporting the adverse effects of *ApoE* ε4 upon brain morphology and cognition in early life comes from animal experiments. Unlike studies in human beings, studies using mice consistently show that, at an early age, *ApoE* ε4 is associated with altered brain biochemistry, reduced dendritic spine density, structural changes to presynaptic and postsynaptic compartments in neurons, and deficits in behavior and memory (Di Battista et al., 2016). The inconsistent conclusions arising from human studies may relate to the numerous compounding factors in human studies along with methodological issues. More studies should be performed to explore the mechanism how *ApoE* polymorphism participates in the physiological and pathological processes of the central nervous system, especially in the growth and development of the central nervous system.

Although more objective and accurate cognitive function and brain morphological measurement methods were employed in this study, the limited sample size of our current study represented a significant limitation, which may be a possible reason for the negative result of the VBM correlation analysis. Although more objective and accurate cognitive function and brain morphological measurement methods were employed herein, the limited sample size of our current study represented a significant limitation, which may be a possible reason for the negative result of the VBM correlation analysis. Although the minimum sample size requirement for VBM analysis was met, a larger sample size would be more favorable to accurately localize atrophic brain regions and explore the brain regions responsible for the cognitive deficits; these need to be determined in future studies. Second, only verbal episodic memory and EF were evaluated in the current study; these parameters cannot fully reflect all differences in cognitive function. In addition, the educational level of our subjects was generally high. Therefore, our conclusions may be biased compared with the overall young population.

In summary, we found that young *ApoE* ε4 carriers had verbal episodic memory and task switching deficits, and reduced GM volume in some brain regions, compared to non-carriers. Our data suggested that the effects of *ApoE* ε4 upon cognition and brain morphology exist long before the possible occurrence of AD.

## Author Contributions

JN performed the research and drafted the manuscript. HS, SM, SZ and XD performed the research. QW, YM and DZ analyzed the data. DZ and XW designed the study and revised the article.

## Conflict of Interest Statement

The authors declare that the research was conducted in the absence of any commercial or financial relationships that could be construed as a potential conflict of interest.

## Footnotes

^1^http://www.fil.ion.ucl.ac.uk/spm

^2^http://www.alivelearn.net/xjview
